# Proanthocyanidins carbon dots inhibit PRRSV infection by activating Nrf2/ARE to regulate oxidative stress and NLRP3 inflammasome-mediated pyroptosis

**DOI:** 10.1186/s13567-025-01642-5

**Published:** 2025-10-16

**Authors:** Fang Wang, Zhiyuan Pan, Fructueux Modeste Amona, Xiaohan Chen, Yipeng Pang, Yuan Liang, Min Lai, Chunlei Zhang, Xi Chen, Xingtang Fang

**Affiliations:** Institute of Cellular and Molecular Biology, School of Life Science, Jiangsu Normal University, Xuzhou, 221000 China

**Keywords:** Proanthocyanidins carbon dots, PRRSV, Nrf2, antiviral, oxidative stress, pyroptosis

## Abstract

Carbon-derived nanomaterials, especially carbon dots (CDs), are gaining attention for their distinct physicochemical characteristics and broad-spectrum antiviral capabilities. However, their efficacy against economically critical pathogens like porcine reproductive and respiratory syndrome virus (PRRSV), a major threat to swine health-and the molecular pathways involved remain underexplored. Here, we developed innovative proanthocyanidin-based carbon dots (PAC-CDs) to enhance their antiviral efficacy against PRRSV by targeting oxidative stress pathways and suppressing NLRP3 inflammasome-mediated pyroptosis via activation of the Nrf2/ARE antioxidant axis. PAC-CD were synthesized as uniformly dispersed spherical nanostructures averaging 5.49 nm in diameter, exhibiting exceptional aqueous solubility and biocompatibility. These nanoparticles significantly suppressed PRRSV replication in both Marc-145 and porcine alveolar macrophage (PAM) cells, while mitigating infection-induced cytopathic damage. Mechanistically, PAC-CDs exert their antiviral effects not through direct viral neutralization but by hindering viral entry and replication via Nrf2 pathway activation, which upregulates antioxidant defenses and reduces oxidative damage. Consistently, PAC-CDs impaired PRRSV-induced NLRP3 inflammasome-triggered pyroptotic cell death and downstream pro-inflammatory cytokine release. These findings advance the development of targeted antiviral therapies and highlight the translational potential of PAC-CDs against PRRSV outbreaks, offering a dual-action strategy to alleviate oxidative injury and inflammatory cascades in infected hosts.

**Supplementary Information:**

The online version contains supplementary material available at 10.1186/s13567-025-01642-5.

## Introduction

Porcine reproductive and respiratory syndrome virus (PRRSV) is an enveloped single-stranded positive-sense RNA virus from the family *Arteriviridae* within the order *Nidovirales* [1]. Over the past 30 years, PRRSV has led to significant economic losses in the global swine industry owing to its high genetic variability and mutations [2]. While vaccination is the primary strategy for the prevention and control of most viral infections, its effectiveness is limited by PRRSV mutagenicity and safety concerns [3]. This has recently paved the way for novel antiviral strategies that address the challenges of untreated viral infection.

Natural compounds have been found to contain multiple bioactive components, which support their potential use in controlling PRRSV infection through various mechanisms [4]. Proanthocyanidins (PAC) are naturally occurring polyphenolic compounds found in various plants. They possess multiple pharmacological activities, including antioxidative, anti-inflammatory, antibacterial, antiviral, immunomodulatory, and antitumor properties [5]. Growing evidence has shown that PAC inhibits viruses such as human papillomavirus (HPV) [6], human immunodeficiency virus (HIV) [7], herpes simplex virus (HSV) [8], coxsackie virus (CV) [9], and hepatitis C virus (HCV) [10]. Further, PAC has been shown to impair the replication and transcription of the SARS-CoV-2 virus by suppressing the activity of its RNA-dependent RNA polymerase (RdRp) [11]. However, owing to their proven bioavailability and stability challenges, nanotechnology has emerged as a promising field with significant potential to overcome these issues and combat infectious diseases [12].

Carbon dots (CDs), or carbon quantum dots (CQDs), are small carbon nanomaterials under 10 nm that have become significant in drug modification due to their excellent properties, including high biocompatibility, low toxicity, good stability, and favorable optical characteristics like fluorescence [13, 14]. Recently, several research studies have shown that CDs exhibit significant antiviral activity against viral infection [15–17]. Further, it has been reported that natural compounds derived from CDs exhibit anti-inflammatory, antioxidant, and antiviral properties against infectious diseases [18, 19]. However, the underlying antiviral mechanism of PAC combined with CDs (PAC-CDs) against PRRSV remains to be elucidated. Furthermore, Nuclear factor-erythroid 2-related factor 2 (Nrf2) is a crucial transcription factor involved in cellular defense against oxidative stress and inflammation by regulating the expression of cytoprotective genes [20]. Under oxidative stress, Nrf2 initiates the transcription of antioxidant enzymes and genes, which reduce oxidative damage [21]. Nrf2 has been shown to support antiviral responses in several viruses, including SARS-CoV-2, HIV-1, Marburg virus, hepatitis B virus (HBV), and respiratory syncytial virus (RSV) [22–26]. Emerging evidence supports that activation of Nrf2 by CDs-derived natural compounds could potentially impair infections [27, 28]. Specifically, Sanjay et al. reported that honeyberry-derived CQDs ameliorate LPS-induced neuroinflammation and oxidative stress through Nrf2/HO-1 signaling in HMC3 cells [29]. Thus, CDs have promising potential, yet green synthesized PAC-CDs that activate Nrf2 to impair viral infections like PRRSV remain to be elucidated.

The NLRP3 inflammasome plays a crucial role in the inflammatory response by attacking recognized pathogens. The apoptosis-associated speck-like protein contains a CARD (ASC) and pro-Caspase-1. Once activated, Caspase-1 cleaves proinflammatory cytokines interleukin-18 (IL-18) and IL-1β, and Gasdermin D (GSDMD), leading to membrane perforation and triggering pyroptosis. This process exacerbates the inflammatory response [27, 30]. PRRSV infection has been reported to induce pyroptosis, promoting the inflammatory response in infected cells and contributing to lung tissue damage [31]. Thus, targeting the inflammasome and pyroptosis-related pathways could alleviate tissue damage caused by PRRSV infection. Moreover, Nrf2 has been shown to negatively regulate NLRP3 inflammasome activation and inhibit pyroptosis [27], thus playing an important role in alleviating inflammatory damage caused by PRRSV infection. So far, multiple studies have shown that Nrf2 inhibits the NLRP3/Caspase-1/GSDMD pathway and pyroptosis by regulating reactive oxygen species (ROS) levels, the thioredoxin system, and the glutathione system, thereby reducing oxidative stress levels [32]. However, to the best of our knowledge, there is no specific research investigating the antiviral activity of PAC-CDs in PRRSV-induced oxidative stress by targeting NLRP3 inflammatory-mediated pyroptosis through Nrf2 activation.

In this study, we synthesized highly biocompatible CDs using PAC as the raw material via a hydrothermal method to investigate the potential antiviral activity of PAC-CDs in PRRSV-induced oxidative stress by targeting NLRP3 inflammasome-mediated pyroptosis via Nrf2 activation. We found that PAC-CDs do not directly inactivate PRRSV but inhibit the virus’s internalization and replication in host cells. Mechanistically, PAC-CDs suppress PRRSV replication by activating the Nrf2/ARE pathway and alleviating virus-induced oxidative stress. Additionally, PAC-CDs reduce inflammation induced by PRRSV infection by inhibiting NLRP3 inflammasome-mediated pyroptosis via Nrf2. These findings suggest that PAC-CDs could potentially be a promising antiviral agent in PRRSV infection.

## Materials and methods

### Cells and viruses

PAMs, the primary target cells of PRRSV in vivo, were prepared from lung lavage fluid of healthy euthanized piglets aged from 4 to 6 weeks. The PAMs were cultured in RPMI 1640 medium (Gibco, New York, USA) supplemented with 10% heat-inactivated fetal bovine serum (FBS) (Gibco), 100 U/mL penicillin, and 100 mg/mL streptomycin (Biosharp). The African green monkey kidney cell line Marc-145 (China Center for Type Culture Collection, China) was maintained in Dulbecco modified Eagle medium (DMEM; SenBeiJia) containing 10% FBS, penicillin, and streptomycin, as described above. All cells were incubated at 37 °C in a humidified atmosphere with 5% CO_2_.

Three North American genotype 2 PRRSV strains were used in this study. The highly pathogenic PRRSV strain BB0907 (GenBank accession number HQ315835.1), referred to as “PRRSV” in this article, was employed in all experiments. Additionally, the classical strain S1 (GenBank accession number DQ459471.1) and the NADC30-like subtype strain FJ1402 (GenBank accession number KX169191.1) were also utilized. The PRRSV strains were propagated in Marc-145 cells and titrated using a 50% tissue culture infective dose (TCID_50_).

### Preparation of PAC and chemicals

PAC is extracted from purple sweet potatoes with a purity of 91%. ML385 was obtained from MedChemExpress (USA). PAC was dissolved in dimethyl sulfoxide (DMSO; Sigma-Aldrich, USA) and diluted with the necessary culture medium before use to retain a final DMSO concentration of less than 0.4%.

### Synthesis of CDs

CDs were synthesized by mixing 3.5 g of PCD (containing 10% salvianolic acid B) with 70 mL of deionized water, as previously described [33]. The mixture was heated in a Teflon-lined stainless-steel autoclave at 150 °C for 6 h, then centrifuged at 10 000 rpm for 10 min to remove large particles. The supernatant was collected and filtered through a 0.22 µm membrane to eliminate smaller particles. Finally, the solution was dialyzed against deionized water using a dialysis bag with a molecular weight cutoff of 14 kDa for 8 h, replacing the deionized water every 2 h to obtain PAC-CDs. The PAC-CDs powder was then collected and freeze-dried for future use.

### Characterization of CDs

Fluorescence measurements were carried out using an RF-5301PC fluorescence spectrophotometer (Shimadzu, Japan). The UV–visible absorption of all samples was measured on a UV-2450 spectrophotometer (Shimadzu). Fourier transform infrared spectroscopy (FTIR) was recorded using a Nicolet Avatar 330 Fourier transform infrared spectrometer (Thermo Fisher Scientific, USA). Size distribution was measured using dynamic light scattering using a Malvern ZetaSizer (Malvern ZEN 3690). X-ray diffraction (XRD) was performed using a D8 Advance X-ray diffractometer (Germany) operating at 40 mA and 40 kV. The surface morphology of the CDs was measured using a JEM-2100F STEM/EDS high-resolution transmission electron microscope (HR-TEM) (JEOL, Japan). Elemental and structural analysis of the carbon dots was characterized using an ESCALAB Xi + X-ray photoelectron spectrometer (XPS) (Thermo Fisher Scientific, USA).

### Cytotoxicity assay

Marc-145 and PAMs reaching 80–90% of confluence were incubated in 96-well plates with different concentrations of PAC and PAC-CDs in DMEM supplemented with 2% FBS for 48 h. Cell viability was assayed according to the instructions of the enhanced CCK8 cell viability kit. Briefly, after replacing the supernatant with 100 μL of fresh DMEM in 96-well plates, 10 μL of CCK8 solution was added to the cells and incubated at 37 °C for 1 h. The optical density (OD) value at 450 nm was measured using an ELISA microplate reader to evaluate the percentage of relative cell viability. The average OD value from six wells per treatment served as the cell viability index, and the 50% cytotoxic concentration (CC_50_) was calculated using GraphPad Prism 5.0 (GraphPad Software, San Diego, California, USA).

### Determination of virus titer

Virus plaque assay: Cells in 6-well plates were cultured until 80% confluence and then infected with tenfold serially diluted PRRSV for 1 h. The culture medium was replaced with DMEM containing 2% low-melting point agarose and 2% FBS. The plates were incubated at 4 °C for 10–20 min to solidify the overlay, then transferred to a cell culture incubator for 2–3 days. Once virus plaques appeared, a neutral red staining solution was added, and the plates were incubated for 1 h. After removing the staining solution, the plates were incubated at 4 °C for approximately 12 h. The numbers of plaques were then counted to calculate the virus titer [34].

TCID_50_: Marc-145 cells growing in 96-well plates were infected with tenfold serially diluted PRRSV. After a 1 h incubation at 37 °C, the medium was replaced with fresh DMEM containing 2% FBS. Virus titer was determined using endpoint dilution analysis at 4 days post-inoculation (dpi). The 50% tissue culture infective dose (TCID_50_) was calculated using the Reed-Muench method [35].

### Real-time quantitative PCR (RT-qPCR) was performed as follows

Total RNA was extracted from cells at specified time points after infection using TRIzol reagent (Tiangen, China). According to the manufacturer’s instructions, 1 µg of total RNA was reverse transcribed into cDNA using the Evo M-MLV Reverse Transcription Kit (Accuratebio, AG11728). RT-qPCR was conducted using the QuantStudio3 system (Applied Biosystems) and SYBR Green qPCR Master Mix (Accuratebio, AG11718). Data were normalized to GAPDH mRNA levels in samples and replicated three times. Relative mRNA expression was calculated using the 2^−ΔΔCt^ method. The RT-qPCR primers used in this study are listed in Additional file 2.

### Western blot analysis

Cells were harvested at specified times after treatment and lysed on ice with cell lysis buffer (Beyotime, P0013B) for at least 20 min. Protein concentrations in samples were quantified. Cell lysates containing equal amounts of protein were subjected to 8–12% sodium dodecyl sulfate–polyacrylamide gel electrophoresis (SDS-PAGE) and electrotransferred to polyvinylidene fluoride (PVDF) membranes (GVS, 1212639). The membranes were blocked in 5% skim milk (Sangon Biotech, China) at room temperature for 1 h to prevent nonspecific binding. After blocking, the membranes were incubated with specific primary antibodies overnight at 4 °C: anti-PRRSV N antibody (GTX637947); anti-Nrf2 (T55136), anti-NQO1 (T56710), anti-NLRP3 (T55651), anti-ASC (PC6741), anti-Caspase1 (M025280), anti-GSDMD (TA4012), and anti-IL-1β (MK56352) from Abmart; anti-β-actin (66,009) and anti-HO-1 (10,701) from Proteintech. Following primary antibody incubation, the membranes were washed three times with TBST buffer. They were then incubated with secondary antibodies, either HRP-conjugated goat anti-mouse IgG polyclonal antibody (HuaAnbio, HA1006) or HRP-conjugated goat anti-rabbit IgG polyclonal antibody (HuaAnbio, HA1001), for 1 h at room temperature. An enhanced chemiluminescence (ECL)-based system was used to visualize immunoreactive proteins (Beyotime, P0018M).

### Indirect immunofluorescence assay (IFA)

PRRSV-infected cells were treated with PAC-CDs at specified concentrations for 48 h and fixed with 4% paraformaldehyde (Biosharp, China) for 10 min. Cells were then permeabilized with 0.5% Triton X-100 (Solarbio, China) in PBS for 15 min. After rinsing with PBS, the cells were blocked with 5% bovine serum albumin (BSA) at room temperature for 1 h and incubated with the antibodies at 4 °C overnight. After washing three times with PBS, they were incubated with the secondary antibodies at room temperature for 1.5 h. The cell nuclei were stained with DAPI (4′,6-diamidino-2-phenylindole dilactate; Beyotime) for 5 min. Images were captured and processed using an inverted fluorescence microscope (U-HGLGPS; Olympus, Japan) or a confocal laser scanning microscope (SP8, Leica, Germany).

### Antiviral activity assay

An antiviral activity assay was conducted to evaluate the effectiveness of PAC-CDs in inhibiting PRRSV in vitro. Monolayers of Marc-145 or PAM cells grown in 96-well or 12-well plates were infected with PRRSV at a multiplicity of infection (MOI) of 0.01 for Marc-145 cells and 0.1 for PAMs, incubated at 37 °C for 1 h. The supernatant was removed, and DMEM (with 2% FBS) containing different concentrations of PAC-CDs was added. Cells and supernatants were then collected post-treatment, and their virus titers were determined by endpoint dilution assay. Viral RNA levels were measured by RT-qPCR, viral N protein levels were analyzed by western blotting, and the number of infected cells was measured by IFA.

### Time‑of‑addition assay

To evaluate which stage of the PRRSV lifecycle is affected by PAC-CDs, a time-of-addition assay was conducted [36], as illustrated in the timeline diagram (Figure 5A). Marc-145 cells seeded in 12-well plates were pretreated, cotreated, or post-treated with PAC-CDs relative to PRRSV inoculation. The experiment was initiated when the cells reached 60% confluence, referred to as -1 h. In the pretreatment group, cells were first treated with PAC-CDs at the specified concentration for 1 h at −1 h, followed by PRRSV infection at 0.01 MOI for 1 h. The supernatant was then replaced with DMEM (containing 2% FBS) for further incubation. In the cotreatment group, cells were treated with PAC-CDs and PRRSV simultaneously for 1 h at 0 h, after which the supernatant was replaced with DMEM (containing 2% FBS). In the post-treatment group, cells were first infected with PRRSV for 1 h at 0 h, and then the medium was replaced with DMEM (2% FBS) containing the specified concentration of PAC-CDs for further incubation. All groups were incubated for 48 h.

### Inactivation assay

PRRSV was incubated with 10 μg/mL PAC-CDs at 37 °C for 1 h. After pre-cooling at 4 °C for 30 min, the pretreated PRRSV was used to infect Marc-145 cells at 4 °C for 1 h, followed by a plaque assay.

### Adsorption assay

Marc-145 cells were pre-cooled at 4 °C for 30 min and then infected with PRRSV at 0.1 MOI in DMEM (containing 2% FBS) with 10 μg/mL PAC-CDs at 4 °C for 1 h. After discarding the supernatant, the cells were washed twice with pre-cooled serum-free DMEM and subjected to a plaque assay.

### Internalization assay

After pre-cooling at 4 °C for 30 min, Marc-145 cells were infected with PRRSV (MOI = 0.1) at 4 °C for 1 h. The supernatant was discarded, and the cells were incubated at 37 °C for 1 h with DMEM (containing 2% FBS) and 10 μg/mL PAC-CDs using pre-cooled serum-free DMEM. Following two washes with serum-free DMEM, a plaque assay was performed.

### Replication assay

Marc-145 cells reached 80–90% confluence in a 24-well plate, and they were infected with PRRSV (MOI = 0.1) at 37 °C for 1 h. The virus inoculum was then removed, and the cells were washed twice with serum-free DMEM to eliminate unadsorbed virus particles, followed by incubation in DMEM (2% FBS) for 6 h. The cells were subsequently incubated with DMEM (containing 2% FBS) and 10 μg/mL PAC-CDs at 37 °C for 7, 8, 9, or 10 h. Total RNA was then isolated, and PRRSV negative-strand RNA was quantified using RT-qPCR.

### Release assay

PRRSV at 0.1 MOI was added to a 24-well plate with a monolayer of Marc-145 cells. After 1 h of adsorption at 37 °C, the cells were washed twice with serum-free DMEM to remove unadsorbed virus particles and then incubated in DMEM (containing 2% FBS) for 18 h. After further washing with serum-free DMEM, the cells were incubated at 37 °C with DMEM (containing 2% FBS) and 10 μg/mL PAC-CDs. Supernatants were collected at 15, 30, 45, or 60 min for plaque assays.

### ROS measurement

ROS levels were measured using a Reactive Oxygen Species Assay Kit (Beyotime Biotechnology, China) according to the manufacturer’s instructions. Marc-145 cells were treated with PRRSV at 1 MOI and varying concentrations (5, 10, 20 μg/mL) of PAC-CDs for 48 h, then incubated with 10 mM DCFH-DA at 37 °C for 30 min. After washing, imaging was performed using an inverted fluorescence microscope (Nikon).

### MDA, GSH, and SOD assays

The levels of superoxide dismutase (SOD), glutathione peroxidase (GSH), and malondialdehyde (MDA) in the cells were measured using SOD Assay Kit (A001-3-2), GSH Assay Kit (A006-2-1), and Microscale MDA Assay Kit (A003-4-1), respectively, according to the manufacturer’s instructions (Nanjing Jiancheng Bioengineering Institute, China). These levels were normalized to protein concentrations determined using a Bicinchoninic Acid (BCA) Protein Assay Kit (Beyotime).

### LDH assay

Lactate dehydrogenase (LDH) levels were assayed using an LDH Assay Kit (Beyotime Biotechnology, C0016). After treating Marc-145 cells with PRRSV (1 MOI) and different concentrations of PAC-CDs (5, 10, 20 μg/mL) for 48 h, LDH levels in the culture medium were measured according to the manufacturer’s instructions.

### Statistical analysis

All statistical analyses were performed using GraphPad Prism 8.0 software. The results are presented as the mean ± standard deviations (SD), with a sample size of at least 3 (*n* ≥ 3). Statistical significance was determined using Student *t*-test for two-group comparisons and one-way analysis of variance (ANOVA) for more than two groups. The values with *P* < 0.05 were considered significant.

## Results

### Synthesis and characterization of PAC-CDs

**Figure 1:**
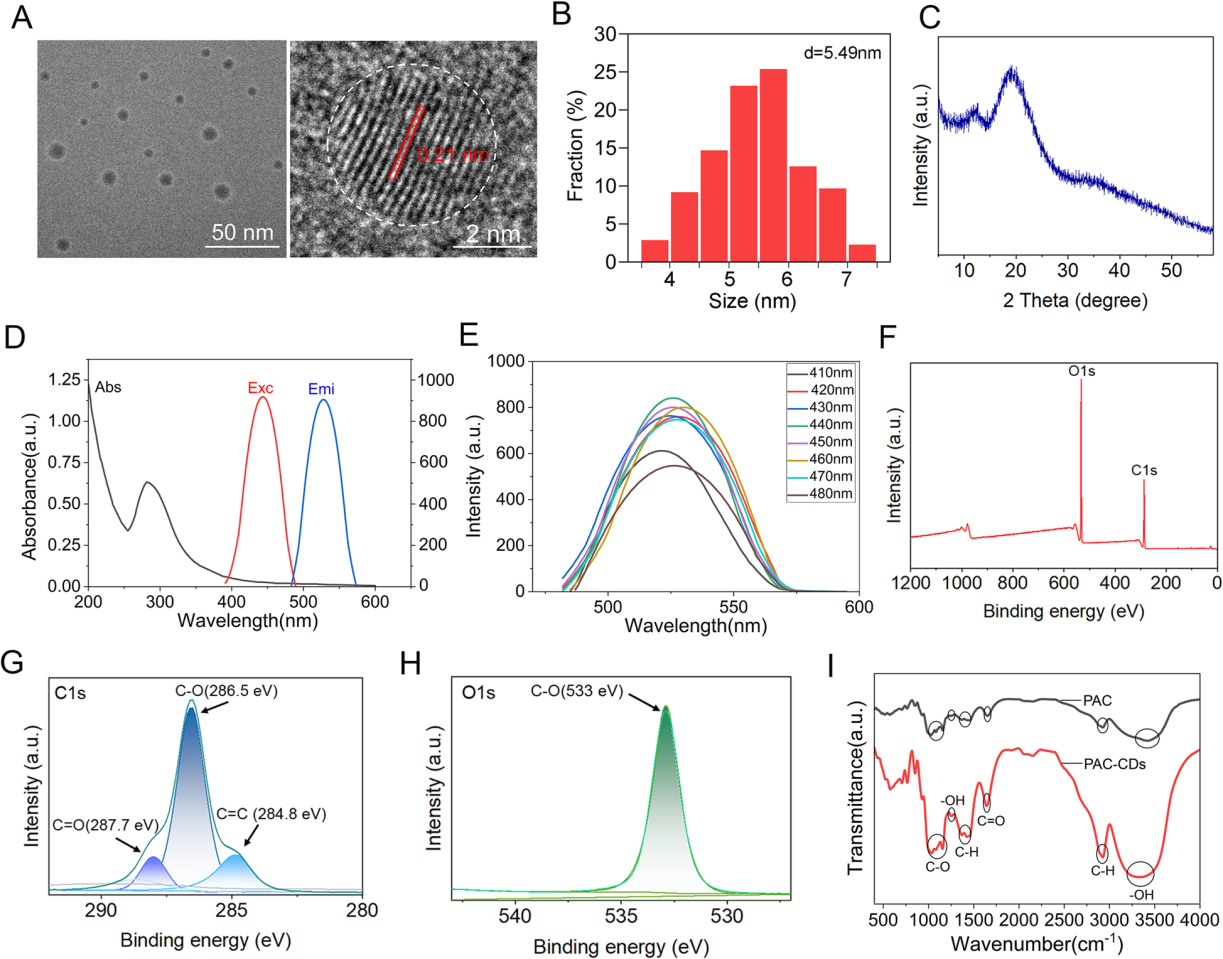
**Synthesis and characterization of PAC-CDs.** **A** TEM image of PAC-CDs. **B** DLS analysis of PAC-CDs. **C** XRD pattern of PAC-CDs. **D** UV–visible absorption (black), fluorescence excitation (red), and emission (blue) spectra of PAC-CDs. **E** FL spectra of PAC-CDs excited at different wavelengths. **F** Full-scan XPS spectrum, **G** high-resolution C1s XPS spectrum, and **H** high-resolution O1s XPS spectrum of PAC-CDs. **I** FTIR spectra of PAC and PAC-CDs.

PAC-CDs powder was obtained through a hydrothermal reaction at 150 °C for 6 h, followed by filtration, centrifugation, and freeze-drying steps. The solid powder appeared brown and was easily soluble in water. The aqueous solution appears yellow, and under UV light irradiation at a wavelength of 365 nm, the aqueous solution of CDs emits strong blue fluorescence (Additional file 1a). Initially, we examined the physicochemical properties of PAC-CDs. Transmission Electron Microscopy (TEM) images revealed a uniform distribution of PAC-CDs with a nearly spherical shape and nanoscale dimensions (Figure 1A). High-resolution TEM images further demonstrated that PAC-CDs exhibit a distinct lattice structure with a lattice spacing of 0.21 nm, which corresponds to (100) lattice planes of graphitic carbon [37]. Dynamic Light Scattering analysis was used to measure the size distribution of PAC-CDs, showing good monodispersity with an average diameter of 5.49 nm (Figure 1B). The X-ray Diffraction (XRD) pattern displayed a broad peak around 20° (Figure 1C), suggesting the presence of amorphous carbon components in PAC-CDs [38]. Furthermore, PAC-CDs exhibited exceptional stability in various physiological solutions, including H_2_O, 0.9% NaCl, PBS, and DMEM medium, with a duration of up to 7 days (Additional file 1B).

UV–vis absorption and fluorescence spectroscopy were utilized to elucidate the optical properties of the PAC-CDs. The UV–vis absorption spectrum shows a prominent absorption peak of approximately 279 nm, attributable to the n-π* transition of the –C = O bond. The fluorescence spectrum reveals that PAC-CDs exhibited characteristic blue fluorescence at 526 nm, with a maximum excitation wavelength maintained at 440 nm (Figure 1D). When the PAC-CDs solution was excited at different wavelengths between 410 and 480 nm (increasing by 10 nm each time), the fluorescence emission spectrum demonstrates pronounced excitation dependency, with variations in both the intensity and position of the emission peak, indicating the excitation-dependent fluorescence emission behavior of the CDs (Figure 1E) [39].

X-ray photoelectron spectroscopy (XPS) was employed to analyze the chemical structure of the CDs. The full-scan XPS spectrum indicates that the CDs were primarily composed of C and O elements, with atomic proportions of 55.98% and 43.72%, respectively (Figure 1F). The high-resolution C1s XPS spectrum exhibits three peaks located at 284.8, 286.5, and 287.7 eV, which were attributed to C = C, C-O, and C = O, respectively (Figure 1G). The peak at 533 eV in the high-resolution O1s spectrum corresponded to C-O (Figure 1H). Fourier transform infrared (FTIR) spectroscopy was conducted to explore the surface functional groups of the CDs. The spectral band at 3452 cm^− 1^ belonged to the stretching vibrations of -COOH and -OH from phenolic hydroxyl groups and carboxylic acids [50]. Characteristic absorption peaks at 2928, 1613, and 1228 cm^− 1^ could be ascribed to the stretching vibrations of C-H, C = O, and –OH, respectively (Figure 1I). The peaks at 1418 and 1355 cm^− 1^ were attributed to the stretching and bending vibrations of C-H, respectively. Based on the characteristic absorption of polysaccharides, the peaks at 1150, 1076, and 1027 cm^− 1^ were due to the stretching vibrations of C-O (Figure 1I) [40]. The comparison of the FTIR spectra indicates that the key functional groups, such as -OH and C = O, are preserved in the PAC-CDs. The FTIR spectrum of PAC-CDs shows characteristic peaks at 3452 cm^− 1^ (–OH and –COOH stretching), 2928 cm^− 1^ (C-H stretching), 1613 cm^− 1^ (C = O stretching), and 1228 cm^− 1^ (C-O stretching). These peaks suggest that functional groups like hydroxyl, carboxyl, and carbonyl are retained during synthesis, similar to those in PAC, but with increased intensity and broader bandwidth, implying that PAC’s functional groups are well-preserved and possibly enhanced in the PAC-CDs spectra.

### PAC-CDs inhibit PRRSV infection in Marc-145 cells

**Figure 2:**
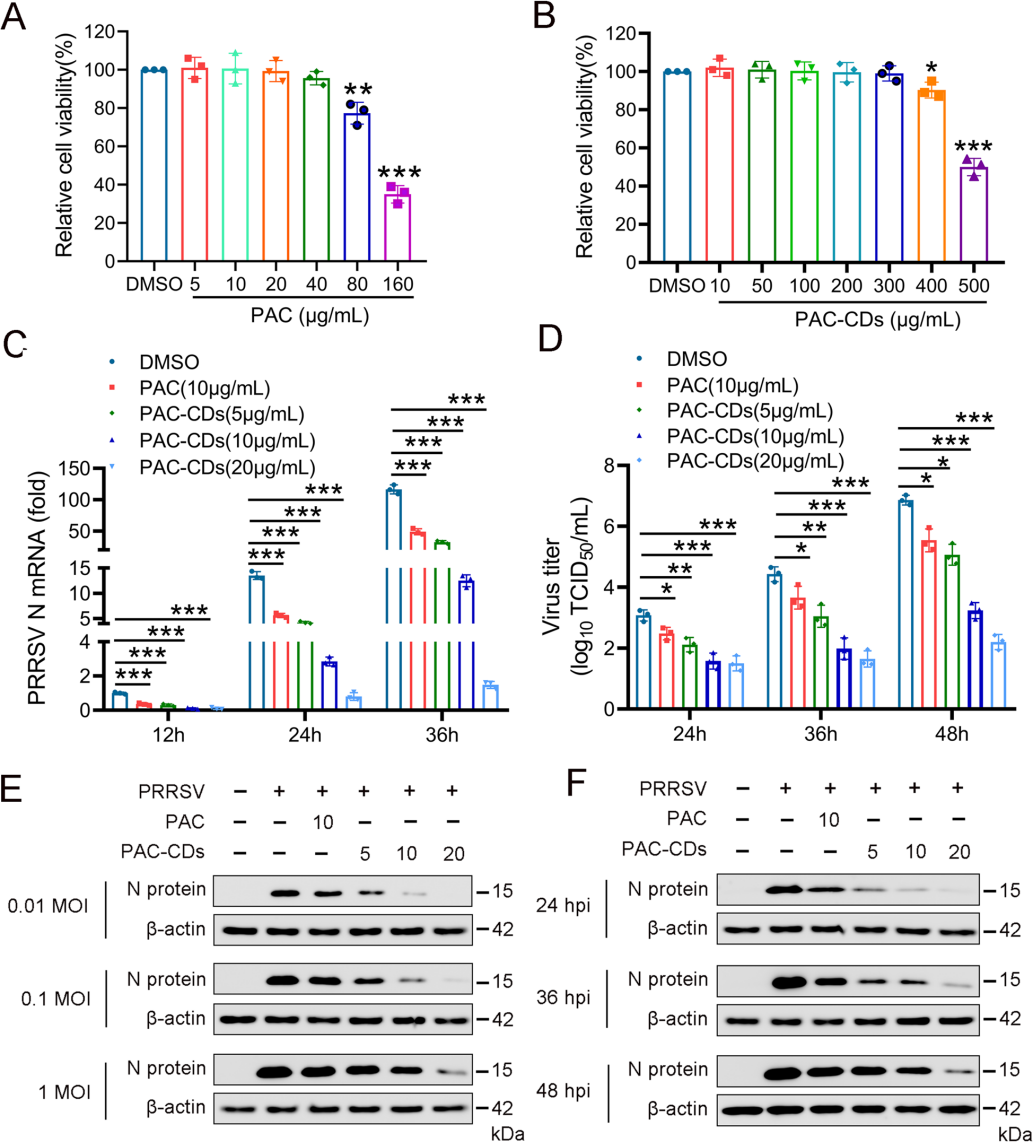
**Cytotoxicity and anti-PRRSV activity of PAC-CDs in Marc-145 cells.** **A**, **B** Cell viability of PAC (**A**) and PAC-CDs (**B**) in Marc-145 cells. **C** Quantification of PRRSV N protein expression at the mRNA level by RT-qPCR in cells treated with DMSO, PAC (10 µg/mL), and PAC-CDs (5, 10, 20 µg/mL) at 12, 24, and 36 hpi. **D** PRRSV titer expressed as log_10_ TCID_50_/mL and measured at 24, 36, and 48 hpi. **E** Western blot analysis shows PRRSV N protein expression in cells infected with PRRSV at different MOI (0.01, 0.1, 1) and treated with PAC or PAC-CDs at 48 hpi. **F** Western blot analysis shows PRRSV N protein expression in cells infected with PRRSV at an MOI of 0.01 and treated with PAC or PAC-CDs at 24, 36, and 48 hpi. β-actin serves as a loading control. PAC served as a control to compare the antiviral activity with PAC-CDs. Data are presented as the mean ± SD of three independent experiments (*P < 0.05, **P < 0.01, ***P < 0.001).

Recent evidence has shown that either PAC or CDs possess antiviral activity against various viruses [41–44]. To investigate the potential antiviral activity of PAC-CDs, we first evaluated the cytotoxicity of PAC and PAC-CDs in Marc-145 cells using the CCK-8 assay. The results show that PAC demonstrate dose-dependent cytotoxicity in Marc-145 cells at concentrations ranging from 80 to 160 μg/mL, with a CC_50_ value of 121.5 μg/mL (Figure 2A). Further, PAC-CDs exhibit dose-dependent cytotoxicity in Marc-145 cells at concentrations between 400 and 500 μg/mL, and the CC_50_ value for PAC-CDs was 495.4 μg/mL (Figure 2B). These results indicate that the cytotoxicity of PAC-CDs is significantly lower than that of PAC. Additionally, we examined whether PAC-CDs inhibit PRRSV infection at different concentrations using RT-qPCR, virus titration, and western blotting. The result shows that PAC-CDs at concentrations between 5 and 20 μg/mL significantly inhibited the mRNA expression of PRRSV N protein in a dose-dependent manner in Marc-145 cells at 12, 24, and 36 h post-infection (hpi) with PRRSV (Figure 2C). Virus titration results indicate that PAC-CDs could reduce PRRSV titers in a dose-dependent manner at 24, 36, and 48 hpi. Compared to the DMSO-treated control, treatment with 20 μg/mL PAC-CDs results in a 4.59 × 10^3^-fold reduction in virus replication at 48 hpi (Figure 2D). Western blot analysis shows that PAC-CDs inhibited PRRSV infection of Marc-145 cells in a dose-dependent manner at MOI of 0.01, 0.1, and 1 (Figure 2E). Similarly, PAC-CDs treatment could significantly reduce the expression level of PRRSV N protein in a dose-dependent manner at 24, 36, and 48 hpi (Figure 2F). Moreover, the antiviral activity of PAC-CDs was higher than that of PAC, whether at the mRNA level, protein level, or virus titer of PRRSV N protein.

**Figure 3:**
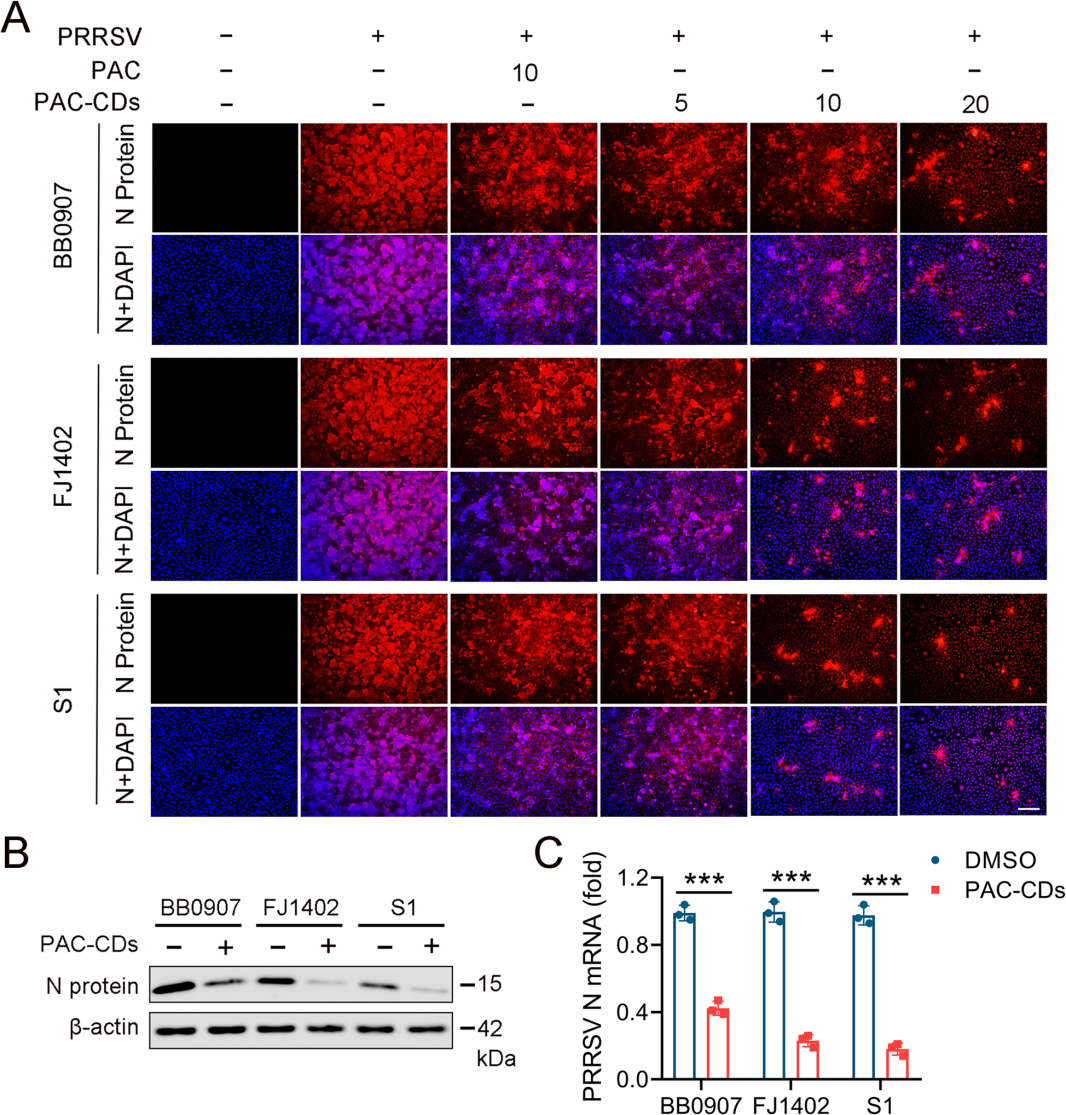
**Antiviral activity of PAC-CDs against different strains of PRRSV.** **A** IFA was used to detect the antiviral activity of PAC-CDs against different strains (BB0907, FJ1402, S1) in Marc-145 cells. Cells grown in 48-well plates were infected with PRRSV (MOI of 0.01) at 37 °C for 1 h and then cultured in fresh media containing different concentrations (5, 10, 20 μg/mL) of PAC-CDs. **B** Expression of viral N protein in cells infected with different strains was detected by western blotting. **C** mRNA expression of viral N protein in cells infected with different strains was detected by RT-qPCR. Scale bar: 100 µm. Data are presented as the mean ± SD of three independent experiments (*P < 0.05, **P < 0.01, ***P < 0.001, ns, non-significant difference).

Next, we investigated the antiviral effect of PAC-CDs on PRRSV strains such as highly virulent strain BB0907, NADC30-like subtype strain FJ1402, and classical strain S1 using indirect IFA at 48 hpi. As shown in Figure 3A, PAC-CDs significantly inhibited the fluorescence intensity of PRRSV N protein in a dose-dependent manner. Similarly, PAC-CDs also significantly inhibited the expression of PRRSV N protein at both the protein and mRNA levels (Figures 3B and C). These results indicate that PAC-CDs effectively inhibit PRRSV infection, and this effect is strain-independent.

### PAC-CDs inhibit PRRSV infection in PAMs

**Figure 4:**
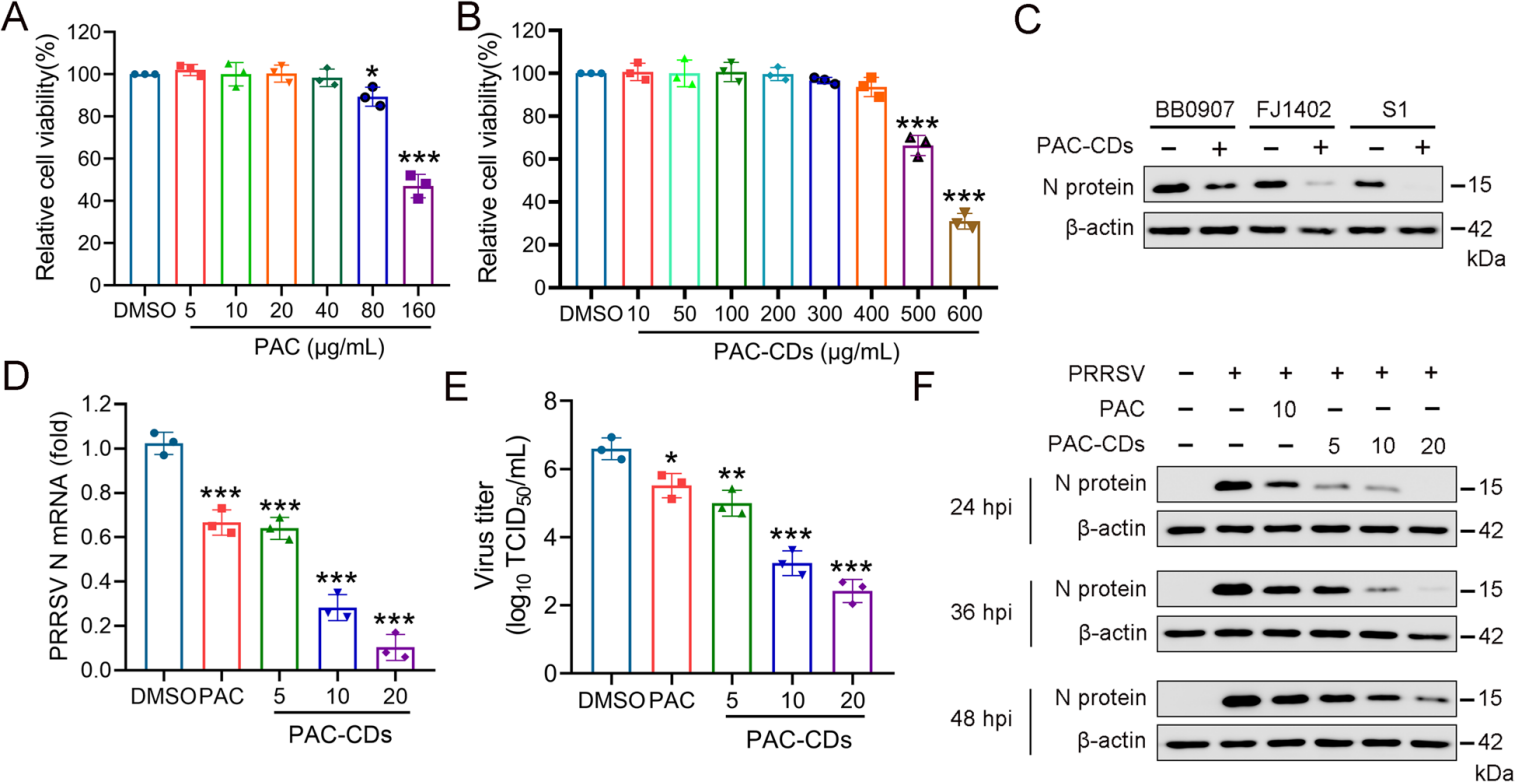
**Inhibition of PRRSV replication in PAMs by PAC-CDs.** **A**, **B** Cell toxicity of PAC and PAC-CDs to PAM was assessed using a CCK8 assay kit. **C** PAMs were infected with different strains (BB0907, FJ1402, S1) of PRRSV for 48 hpi, and viral N protein expression in the cells was detected by western blotting. **D** PAMs were treated with PAC-CDs at different concentrations (5, 10, 20 μg/mL) for 36 h, and the mRNA expression of viral N protein was detected by RT-qPCR. **E** PAMs were treated with PAC-CDs at different concentrations for 48 h, and PRRSV titers were determined using an endpoint dilution assay. **F** PAMs were treated with PAC-CDs at different concentrations, and viral N protein expression was detected by western blotting at 24, 36, and 48 hpi. Data are presented as the mean ± SD of three independent experiments (*P < 0.05, **P < 0.01, ***P < 0.001).

PAMs are the primary target cells for PRRSV infection in pigs [45], and we investigated whether PAC-CDs could inhibit PRRSV in these cells. Initially, we assessed the cytotoxicity of PAC and PAC-CDs to PAMs using the CCK8 assay. As shown in Figure 4A, PAC exhibited significant cytotoxicity at concentrations of 80 μg/mL and 160 μg/mL, with a CC_50_ value of 146.8 μg/mL. PAC-CDs, however, demonstrates notable cytotoxicity at concentrations of 500 μg/mL and 600 μg/mL, with a CC_50_ value of 563.8 μg/mL (Figure 4B). Notably, the toxicity of PAC-CDs to PAMs was significantly lower than that of PAC, and PAMs show higher tolerance than Marc-145 cells.

Next, we investigated the antiviral activity of PAC-CDs in PAMs-infected cells using RT-qPCR, virus titration, and western blot analyses. The result shows that PAC-CDs significantly inhibited the expression of the N protein for all three virus strains (Figure 4C). Additionally, PAC-CDs treatment resulted in a dose-dependent reduction in viral mRNA levels, protein levels, and virus titers (Figures 4D, E, F). Importantly, PAC-CDs demonstrate higher antiviral activity compared to PAC. Taken together, these findings indicate that PAC-CDs effectively inhibit PRRSV infection in PAMs.

### PAC-CDs inhibit PRRSV infection under different treatment modes

**Figure 5:**
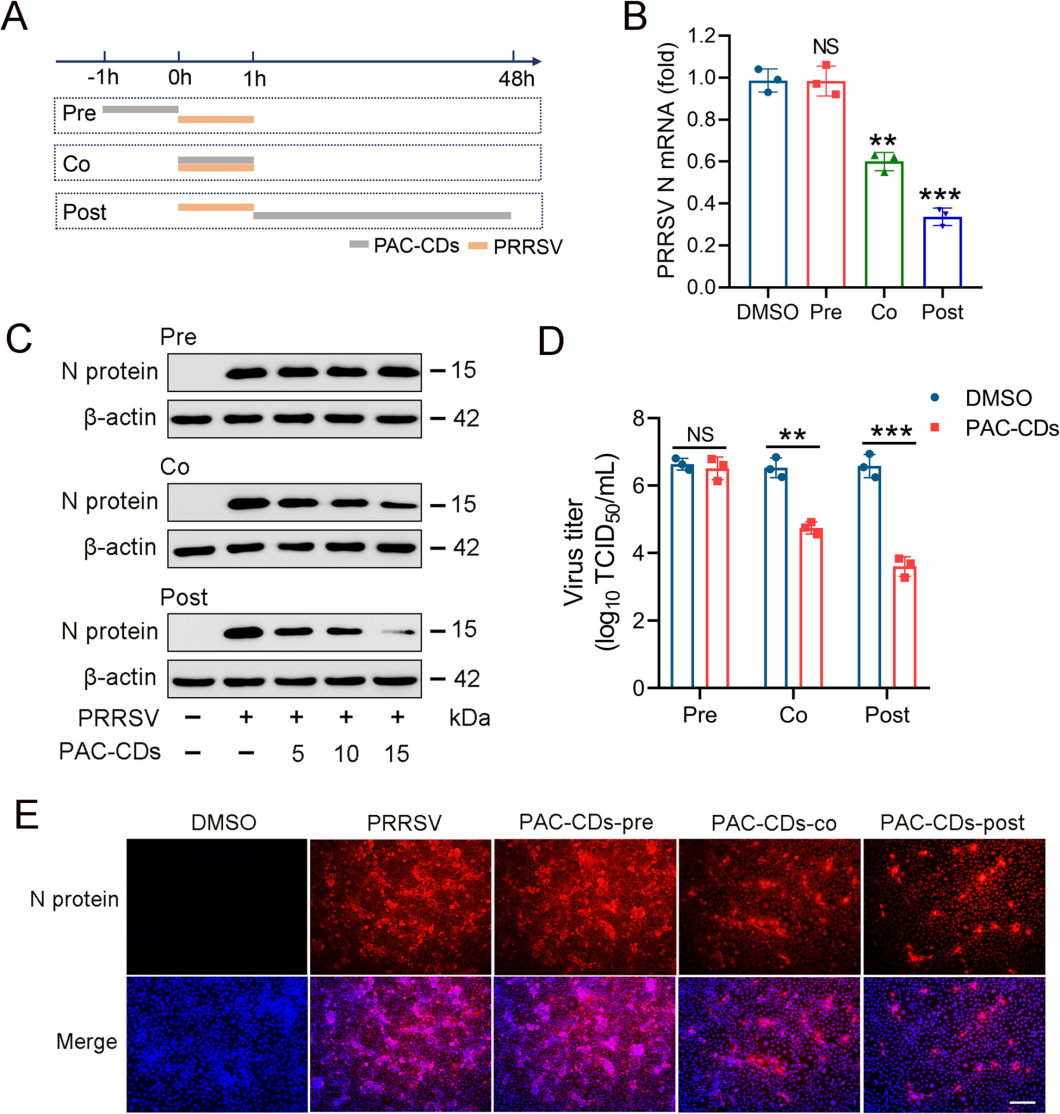
**Effects of PAC-CDs on PRRSV replication in Marc-145 cells in pretreatment, cotreatment, and post-treatment modes.** **A** Schematic diagram of the addition time. Marc-145 cells were infected with PRRSV (0.01 MOI) for 1 h and treated with PAC-CDs at specified concentrations at different times of infection, designated as pretreatment (pre), cotreatment (co), or post-treatment (post). At 48 hpi, cells and supernatants were harvested for RT-qPCR **B**, western blot **C**, virus titer **D**, and IFA **E** analysis. Scale bar: 100 µm. Data are presented as the mean ± SD of three independent experiments (*P < 0.05, **P < 0.01, ***P < 0.001, ns, non-significant difference).

To explore when PAC-CDs exert their antiviral effects during the PRRSV replication cycle, we assessed the time of addition. PAC-CDs were added to cell cultures before (pre-treatment), during (co-treatment), and after (post-treatment) PRRSV infection (Figure 5A). Detection methods included RT-qPCR, western blot, virus titer assay, and IFA. As shown in Figures 5B-E, treating cells with PAC-CDs for 1 h before infection had no significant effect on PRRSV replication. However, treatment during and after infection significantly inhibited PRRSV replication, with post-treatment showing a greater inhibitory effect than co-treatment. Taken together, these results indicate that treating Marc-145 cells with PAC-CDs during or immediately after virus infection significantly inhibits PRRSV replication.

### PAC-CDs inhibit PRRSV internalization and replication

**Figure 6:**
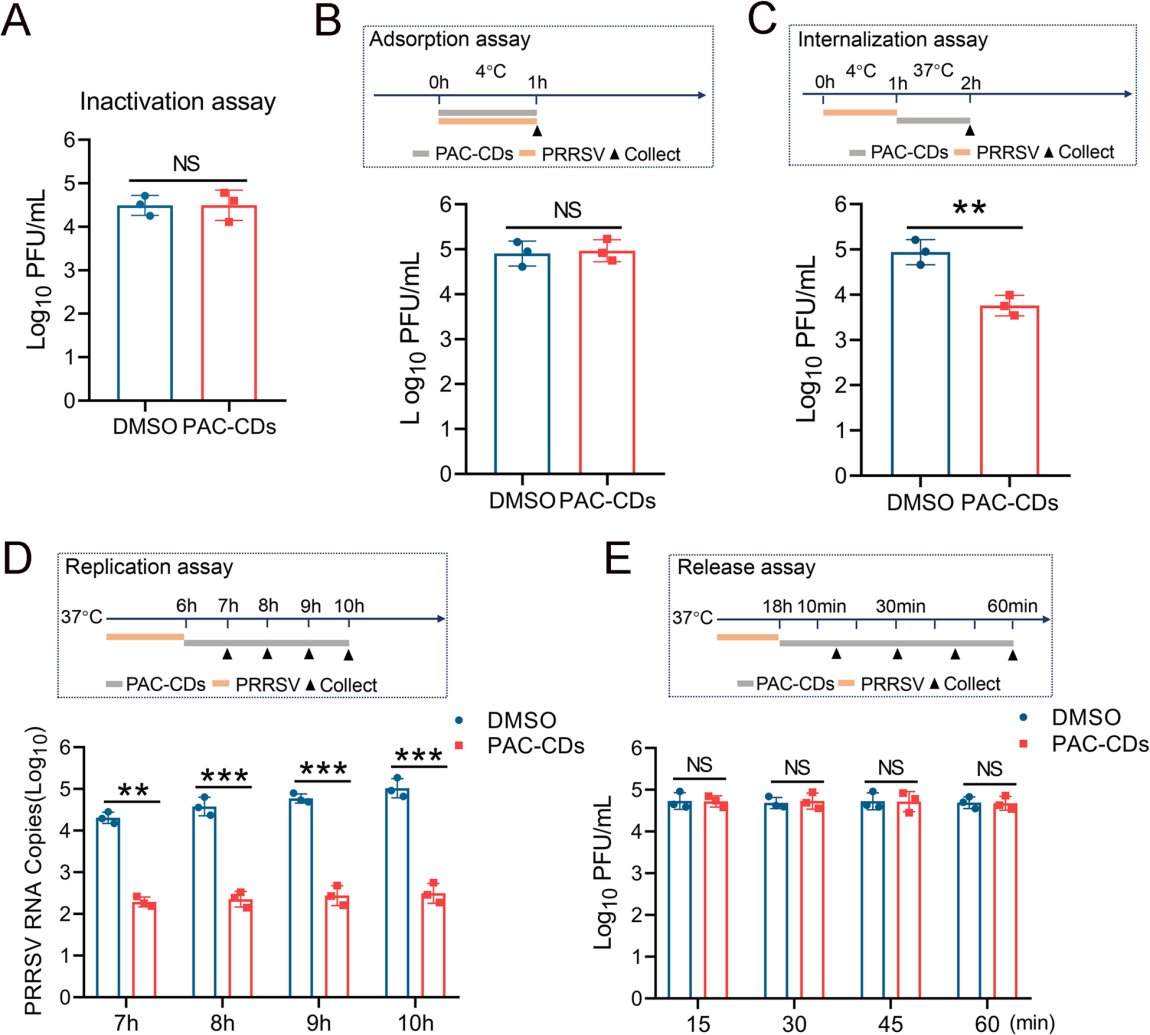
**Multiple-stage inhibition of PAC-CDs on PRRSV proliferation.** **A** Effect of PAC-CDs on direct inactivation of PRRSV. The effect of PAC-CDs on the infectivity of Marc-145 cells on the adsorption **B**, internalization **C**, replication **D**, and release **E** processes of PRRSV. Data are presented as the mean ± SD of three independent experiments (*P < 0.05, **P < 0.01, ***P < 0.001, ns, non-significant difference).

To investigate the mechanism of PAC-CDs’ antiviral effect, we analyzed their impact on PRRSV proliferation during various stages: adsorption, internalization, replication, and release. Firstly, we tested whether PAC-CDs could directly inactivate PRRSV. Plaque assays show that PAC-CDs did not directly inactivate PRRSV (Figure 6A). During the adsorption phase, there was no significant difference between the PAC-CDs and the control group in inhibiting PRRSV adsorption (Figure 6B). In the internalization process, treatment with PAC-CDs reduced the infectious virus titer by tenfold compared to the control, indicating they impede virus internalization (Figure 6C).

To evaluate the effect of PAC-CDs on virus replication, PRRSV negative-strand RNA levels were analyzed by RT-qPCR. The result shows that PAC-CDs reduced PRRSV RNA copies by 10^3^-fold (Figure 6D), suggesting their primary mechanism of action is by suppressing replication. Additionally, we examined the impact of PAC-CDs on the release of PRRSV progeny. In Figure 6E, no significant difference was observed in the virus titer between the PAC-CDs group and the control group, suggesting that PAC-CDs do not inhibit the release of PRRSV progeny. In summary, PAC-CDs inhibit PRRSV proliferation by targeting the internalization and replication processes of PRRSV, but they have no inhibitory effect on the adsorption and release of progeny viruses.

### PAC-CDs exert antiviral effects and inhibit PRRSV-induced oxidative stress by activating the Nrf2/ARE pathway

**Figure 7:**
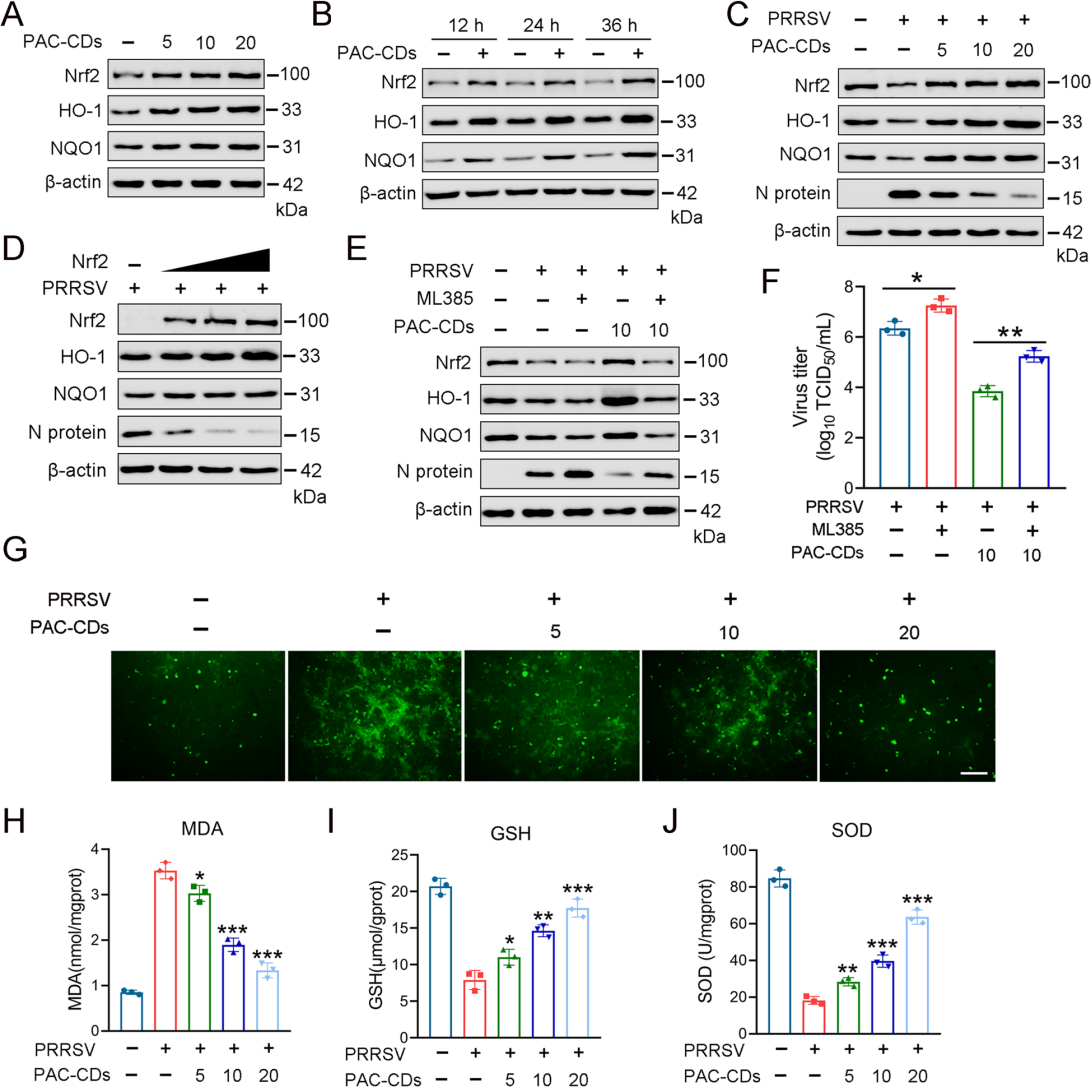
**PAC-CDs exert antiviral effects and suppress PRRSV-induced oxidative stress by activating the Nrf2/ARE signaling pathway.** **A** Western blotting shows the effect of PAC-CDs on the Nrf2/ARE pathway after Marc-145 cells were treated with different concentrations (5, 10, and 20 μg/mL) of PAC-CDs for 36 h. **B** Effects on Nrf2/ARE pathway after PAC-CDs treatment at 12, 24, and 36 h. **C** Effect of PAC-CDs on Nrf2/ARE pathway in Marc-145 cells infected with PRRSV. **D** Effect of Nrf2 overexpression plasmid on PRRSV replication. **E** Effect on viral replication was detected by protein blotting after treating cells with 5 μM ML385 for 48 h. **F** Titer of PRRSV was determined by end-point dilution after treating cells with 5 μM ML385 for 48 h. **G**–**J** PRRSV infected Marc-145 cells were treated with different concentrations of PAC-CDs for 48 h. The levels of ROS, MDA, GSH and SOD were measured. Scale bar: 100 µm. Data are presented as the mean ± SD of three independent experiments (*P < 0.05, **P < 0.01, ***P < 0.001).

Oxidative stress, which disrupts the body’s redox balance, is a significant pathogenic mechanism of viral infection [46]. Nrf2 regulates the expression of various antioxidants and helps provide resistance to oxidative stress [26]. Therefore, we investigated whether PAC-CDs antagonism against PRRSV infection is related to the Nrf2 antioxidant pathway. We treated uninfected Marc-145 cells with different concentrations of PAC-CDs for 12, 24, and 36 h, respectively. The western blot results show that PAC-CDs could dose-dependently increase the protein expression of Nrf2, HO-1, and NQO1 (Figure 7A). Similarly, with increasing treatment time, PAC-CDs gradually upregulated the protein expression of Nrf2, HO-1, and NQO1 in uninfected cells (Figure 7B). Furthermore, when PAC-CDs were used to treat PRRSV-infected Marc-145 cells, the expression levels of Nrf2, HO-1, and NQO1 were increased compared to the PRRSV-infected group without PAC-CDs treatment (Figure 7C). Collectively, these results demonstrate that PAC-CDs can activate the Nrf2/ARE pathway in both uninfected and infected cells.

To investigate whether PAC-CDs exert antiviral effects by upregulating Nrf2, we transfected Marc-145 cells with plasmids that overexpress Nrf2, and the cells were infected with PRRSV for 24 h. The results show that cells overexpressing Nrf2 exhibited resistance to PRRSV replication in a dose-dependent manner (Figure 7D), indicating that Nrf2 is an antiviral factor. When Marc-145 cells were treated with the Nrf2 inhibitor ML385, it promoted PRRSV replication and reversed the antiviral effects of PAC-CDs (Figure 7E). By measuring the virus titer in the cell supernatant, we found that compared to the PAC-CDs-treated group, ML385 could reverse the inhibitory effect of PAC-CDs on the virus titer (Figure 7F).

CDs have been shown to effectively regulate intracellular ROS, helping to maintain redox balance and reduce the production of inflammatory mediators [47]. This regulation is crucial as ROS plays a significant role in signaling pathways that can exacerbate inflammatory conditions [48]. Thus, to explore the impact of PAC-CDs on oxidative stress induced by PRRSV, we determined the effects of PAC-CDs on the levels of ROS, MDA, GSH, and SOD in PRRSV-infected cells. The results show that, compared with the PRRSV-infected group, PAC-CDs significantly reduced the levels of ROS induced by PRRSV (Figure 7G). Meanwhile, MDA, a biomarker related to lipid peroxidation and oxidative stress, was significantly increased in the PRRSV-infected cells, while PAC-CDs treatment significantly reduced MDA levels in PRRSV-infected cells (Figure 7H). Furthermore, PRRSV infection decreased the expression of the antioxidant enzymes GSH and SOD, whereas PAC-CDs treatment significantly increased their expression levels (Figures 7I, J). These results suggest that PAC-CDs can alleviate oxidative damage in Marc-145 cells caused by PRRSV infection to some extent.

### PAC-CDs suppress NLRP3-mediated pyroptosis and reduce the expression of inflammatory cytokines by activating Nrf2

**Figure 8:**
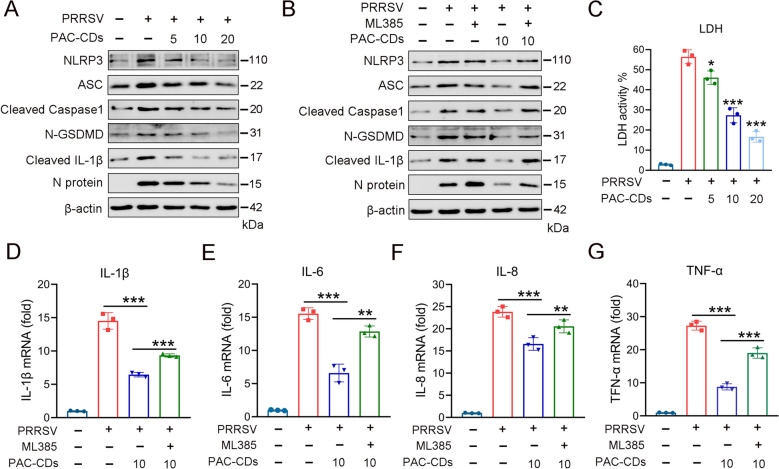
**PAC-CDs inhibit NLRP3-mediated pyroptosis and reduce the expression of inflammatory cytokines.** **A**, **B** Western blotting shows protein levels of pyroptosis-related proteins after treatment with PAC-CDs at different concentrations of 5, 10, and 20 μg/mL (A) and (B) ML385 reverses the effects on pyroptosis. **C** LDH content was measured by a microplate reader after PAC-CDs treatment. **D**–**G** Effects of PRRSV and ML385 on inflammatory cytokines measured by RT-qPCR. After transfection with PAC-CDs and ML385 for 48 h, the mRNA levels of inflammatory cytokines IL-1β, IL-6, IL-8, and TNF-α. Data are presented as the mean ± SD of three independent experiments (*P < 0.05, **P < 0.01, ***P < 0.001).

The NLRP3 inflammasome is crucial for innate immune responses and has gained significant attention regarding viral infections [49, 50]. Recent studies have shown that various viruses can activate or inhibit the NLRP3 inflammasome through their viral particles, proteins, and nucleic acids [33, 51]. To investigate the effects of PAC-CDs on NLRP3 inflammasome-mediated pyroptosis, we assessed the effects of PAC-CDs treatment on proteins related to the pyroptosis pathway, including NLRP3, ASC, Caspase1, GSDMD, and IL-1β. The results show that PRRSV increased the expression levels of NLRP3, ASC, Cleaved Caspase1, N-GSDMD, and cleaved IL-1β, while PAC-CDs treatment dose-dependently decreased the expression of these pyroptosis-related proteins (Figure 8A), indicating that PAC-CDs can inhibit PRRSV-induced pyroptosis. Studies have shown that pyroptosis can damage the cell membrane, leading to the release of LDH from the cell [52]. Thus, we investigated the effect of PAC-CDs on LDH release. The results demonstrate that PRRSV infection increased LDH release, while PAC-CDs treatment dose-dependently inhibited LDH release, thereby alleviating virus-induced pyroptosis (Figure 8C).

Furthermore, it has been reported that Nrf2 negatively regulates the NLRP3 inflammasome-mediated pyroptosis by inhibiting the activity of ROS and modulating signaling pathways [53]. This regulation helps to protect cells from inflammation and cell death associated with the pyroptotic process. Thus, we explored whether PAC-CDs suppress PRRSV-induced pyroptosis through Nrf2, thereby mitigating inflammatory responses. Compared to the PAC-CDs group, ML385 treatment increased the expression levels of NLRP3, ASC, Cleaved Caspase1, N-GSDMD, and Cleaved IL-1β, promoting the occurrence of pyroptosis (Figure 8B). This indicates that ML385 can reverse the inhibitory effect of PAC-CDs on pyroptosis. Next, we used RT-qPCR to assess the impact of PAC-CDs on inflammatory cytokines. We found that compared to the PRRSV-infected group, PAC-CDs treatment reduced the expression levels of the inflammatory cytokines IL-1β, IL-6, IL-8, and TNF-α. However, compared to the PAC-CDs group, ML385 increased the expression of these inflammatory cytokines (Figures 8D-G). These data suggest that PAC-CDs activated the Nrf2 pathway, suppressing PRRSV-induced pyroptosis and the expression of inflammatory cytokines.

### PAC-CDs suppress PRRSV replication in PAMs by activating the Nrf2 pathway, thereby mitigating cell pyroptosis and inflammation

**Figure 9:**
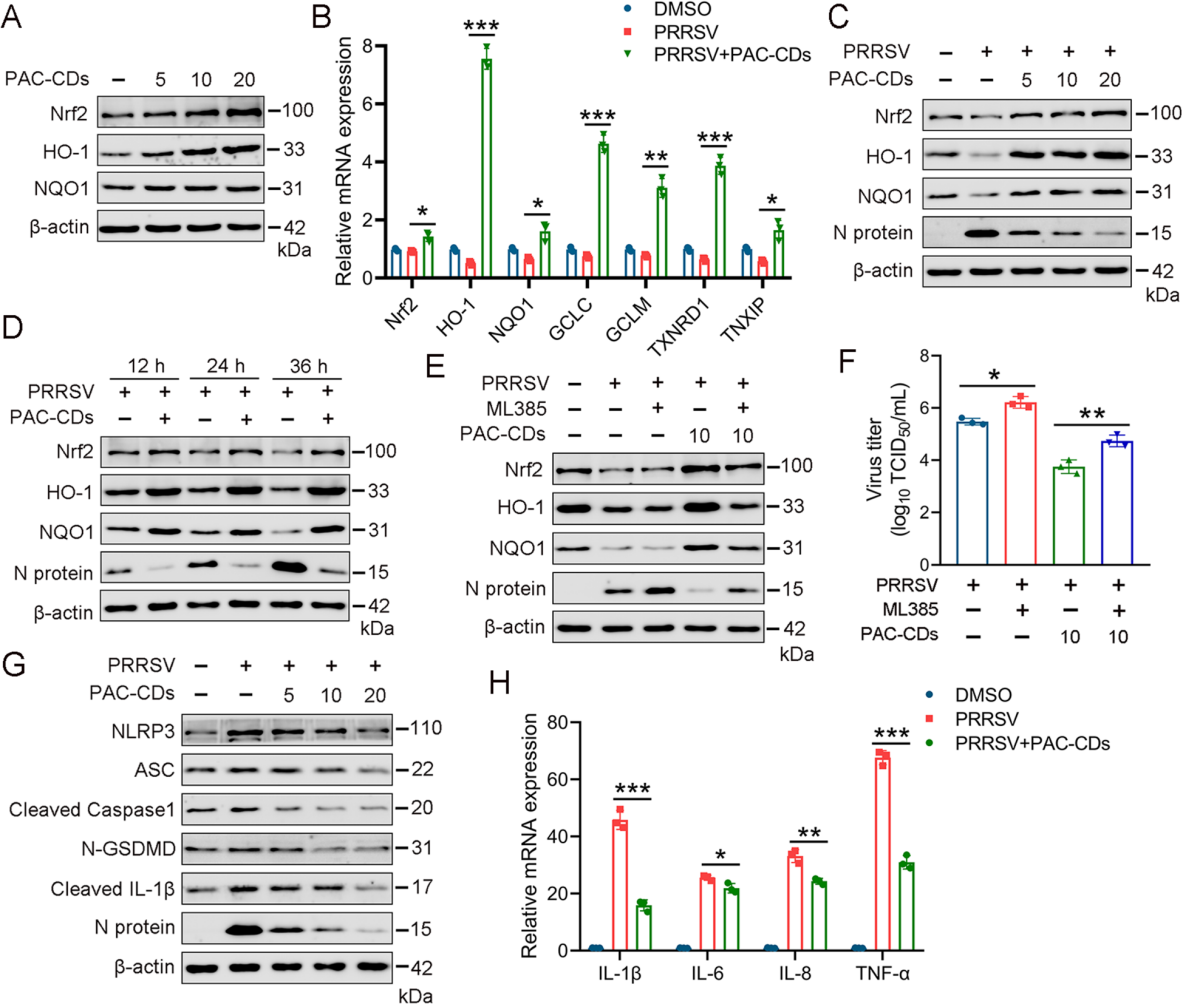
**PAC-CDs suppress PRRSV replication in PAMs by activating the Nrf2 pathway to alleviate pyroptosis and inflammation.** **A** and **C** Western blotting shows the expression of the Nrf2/ARE pathway after infected and uninfected PAMs were treated with PAC-CDs at different concentrations (5, 10, 20 μg/mL). **B** RT-qPCR shows the mRNA expression of the Nrf2/ARE pathway after PAMs infected with PRRSV were treated with PAC-CDs at different concentrations. **D** Protein expressions of the Nrf2/ARE pathway in the cells were detected by western blotting at 12, 24, and 36 h after Marc-145 cells were infected with PRRSV and treated with PAC-CDs. **E** Western blotting shows the protein expression of the Nrf2/ARE pathway and N proteins after treating PAMs with 5 μM ML385 for 36 h. **F** Titer of PRRSV was determined using an endpoint dilution method after treating PAMs with 5 μM ML385 for 36 h. **G** Western blotting shows the expression of pyroptosis-related proteins after PAMs infected with PRRSV were treated with PAC-CDs at different concentrations. **H** The mRNA levels of inflammatory cytokines IL-1β, IL-6, IL-8, and TNF-α were detected by RT-qPCR at 36 h after PAMs infected with PRRSV were treated with PAC-CDs. Data are presented as the mean ± SD of three independent experiments (*P < 0.05, **P < 0.01, ***P < 0.001).

Given that PAMs (porcine alveolar macrophages) are the primary target cells for PRRSV infection in pigs [45], we investigated whether PAC-CDs could inhibit PRRSV replication in PAMs by upregulating Nrf2 to alleviate virus-induced cell pyroptosis and inflammation. PAMs infected and uninfected with PRRSV were treated with PAC-CDs. Protein expression levels were assessed using western blotting. The result shows that PAC-CDs increased the expression of Nrf2, HO-1, and NQO1 in uninfected cells (Figure 9A). Similarly, in PAMs infected with PRRSV, PAC-CDs induced the expression of Nrf2, HO-1, and NQO1 in a dose- and time-dependent manner and suppressed PRRSV replication (Figures 9C, D). PAC-CDs significantly upregulated the mRNA expression level of Nrf2 and its downstream targets, including HO-1, NQO1, GCLM, GCLC, TXNRD1, and TNXIP (Figure 9B). However, ML385 reduced the protein expression levels of Nrf2, HO-1, and NQO1 induced by PAC-CDs while increasing N protein expression (Figure 9E). Additionally, ML385 reversed the inhibitory effect of PAC-CDs on virus titer (Figure 9F). These results indicate that PAC-CDs suppress PRRSV replication in PAMs by activating the Nrf2/ARE pathway.

Furthermore, we investigated the effects of PAC-CDs on pyroptosis and inflammation in PAMs. We found that PAC-CDs reduced the expression levels of NLRP3, ASC, Cleaved Caspase1, N-GSDMD, and cleaved IL-1β in PAMs in a dose-dependent manner, alleviating PRRSV-induced cell pyroptosis (Figure 9G). RT-qPCR analysis shows that PAC-CDs reduced the mRNA levels of inflammatory cytokines IL-1β, IL-6, IL-8, and TNF-α, thereby suppressing inflammation in PAMs (Figure 9H). These findings demonstrate that PAC-CDs can reduce cell pyroptosis and inhibit inflammatory cytokine expression, ultimately improving the inflammatory status of cells.

## Discussion

Currently, there is no effective treatment for PRRSV in clinical settings, and existing prevention strategies, including inactivated and attenuated vaccines, do not effectively halt the disease’s occurrence and spread [54]. Furthermore, vaccination may increase the host’s susceptibility to secondary infections from other viruses. This condition, known as vaccine-induced enhanced susceptibility, has been reported in lentivirus infections, where the host may become more vulnerable after vaccination [55]. Hence, developing anti-PRRSV drugs is crucial for clinical application. Recent studies indicated that natural compounds derived from carbon dots may offer therapeutic benefits to mitigate the negative effects of oxidative stress during viral infections [17–19]. In this study, we synthesized novel carbon dots exhibiting antiviral, anti-inflammatory, and antioxidant properties via a hydrothermal reaction at 150 °C for 6 h, utilizing PAC as precursors. PAC-CDs exhibit good water solubility and extremely low cytotoxicity. TEM results revealed that the carbon dots are uniformly sized and possess a distinct lattice structure. Their nanoscale size enhances cellular internalization and therapeutic efficacy. FTIR and XPS analysis show that PAC-CDs are primarily composed of elements such as carbon and oxygen, with a surface rich in functional groups, including –OH, –COOH, and C = O. These properties endow PAC-CDs with excellent chemical and biological performance. These results agreed with previous research, where carbon-based nanomaterials have been characterized in viral infection [27, 56].

Furthermore, PAC-CDs, with an average diameter of 5.49 nm, significantly inhibited PRRSV replication in both Marc-145 cells and PAMs, achieving a 4.59 × 10^3^-fold reduction in progeny virus production at 48 hpi, markedly surpassing the efficacy of PAC alone [42]. Crucially, PAC-CDs exhibited significantly lower cytotoxicity (CC₅₀ ≈ 495 μg/mL in Marc-145 cells) compared to native PAC (CC₅₀ ≈ 121.5 μg/mL), attributable to their distinct physicochemical properties arising from surface modification during carbonization. The transformation of PAC into carbon dots introduces a hydrophilic surface rich in oxygen-containing functional groups (–OH, C = O, –COOH; Figures 1G-I), which enhances aqueous solubility and reduces non-specific hydrophobic interactions with cellular membranes-a key driver of PAC’s inherent cytotoxicity [13, 38]. This surface modification effectively “shields” PAC’s cytotoxic moieties within the carbon core while preserving its bioactive polyphenolic structure, as evidenced by retained FTIR peaks (Figure 1I). The nanoscale architecture (5.49 nm) and spherical morphology (Figure 1A) further contribute to biocompatibility by enabling efficient cellular internalization via endocytic pathways, minimizing membrane disruption [43, 57]. Consequently, PAC-CDs achieve a higher therapeutic index (CC₅₀/EC₅₀), allowing administration of effective antiviral doses (20 μg/mL) far below cytotoxic thresholds.

While PAC alone inhibits PRRSV through multi-stage interference (adsorption, internalization, replication, release) and direct virucidal activity [42], PAC-CDs specifically target internalization and replication, likely due to their surface-driven interactions with viral entry machinery and enhanced intracellular delivery to replication sites. The larger surface area and multivalent binding capacity of PAC-CDs [57, 58] amplify these effects, explaining their superior potency (4.59 × 10^3^-fold titer reduction vs. PAC’s tenfold). Thus, the shift from PAC to PAC-CDs transition fundamentally alters the compound’s bio-nano interface, optimizing both safety and efficacy against PRRSV. Similarly, several studies indicated that the size, charge, and surface modification of carbon nanomaterials significantly impact their antiviral activity [17, 44, 59]. Ting et al. synthesized curcumin carbon dots (CCM-CDs) from curcumin and citric acid using a hydrothermal method. They found that the positively charged CCM-CDs can alter the structure of the virus surface through electrostatic interactions, inhibiting Porcine epidemic diarrhea virus (PEDV) internalization into Vero cells and reducing virus replication [60]. Similarly, Tong et al. prepared glycyrrhizic acid carbon dots (Gly-CDs) using glycyrrhizic acid, which showed a fivefold increase in inhibitory activity against PRRSV in Marc-145 cells compared to glycyrrhizic acid alone. Further investigation revealed that Gly-CDs inhibit PRRSV by direct inactivation and target the internalization and replication stages [43]. Virus infection of cells is an extremely complex dynamic process involving changes in virus structure, cellular immune response, and alterations in the virus-hosting cell microecology. Therefore, it is essential to comprehensively explore the mechanism of action of PAC-CDs from multiple perspectives.

Natural compounds can exert a virucidal effect by binding to viruses and can also inhibit viral proliferation by acting on different stages of viral infection, including adsorption, internalization, replication, assembly, and release [61]. Xanthohumol acts on the adsorption and internalization stages of PRRSV [62], while curcumin affects the internalization stage of PRRSV but cannot inactivate the virus [63]. Additionally, carbonization may alter the antiviral mechanism of the synthesized carbon dots. Glycyrrhizic acid have been reported to inhibit the internalization stage of PRRSV without significant effects on its adsorption, replication, or release stages. However, Gly-CDs, synthesized primarily from glycyrrhizic acid, can inhibit not only the internalization but also the replication stage of PRRSV and have a virucidal effect on PRRSV [43]. Previous studies have shown that PAC can simultaneously inhibit the adsorption, internalization, replication, and release stages of PRRSV and has a virucidal effect on PRRSV [42]. Consistent with these findings, we found that PAC-CDs can inhibit the internalization and replication stages of PRRSV but cannot inactivate the virus, indicating that the antiviral mechanisms of PAC-CDs and PAC are different.

Moreover, oxidative stress plays a significant role in viral infections by contributing to reduced antiviral host responses and increasing inflammation and pyroptosis that ultimately drive cell and tissue damage [64]. Viruses can explore mitochondrial functions to induce oxidative stress, which may further impair the immune response [64]. The upregulation of ROS is associated with mitochondrial dysfunction [65]. Research has shown that ROS generation is commonly observed in virus-infected cells. Influenza virus infection elevates the formation of reactive peroxynitrite and induces oxidative stress in the lungs [66], while PRRSV infection triggers oxidative stress through ROS production in Marc-145 cells [67]. Dengue virus infection significantly increases cellular oxidative stress in dendritic cells [68]. PRRSV downregulates the expression of superoxide dismutase, reduces glutathione levels, and increases the production of ROS and malondialdehyde in Marc-145 cells, PAMs, and the lung tissues of infected pigs [69]. Consistently, this study revealed that PAC-CDs treatment effectively mitigates PRRSV-induced oxidative stress. PAC-CDs can upregulate the expression of Nrf2, HO-1, and NQO1 in Marc-145 and PAMs, thereby exerting antiviral effects. However, Nrf2 inhibitors can reverse the antiviral effects of PAC-CDs, suggesting that PAC-CDs induce the activation of the Nrf2 signaling pathway to mitigate PRRSV infection. The Nrf2-mediated antioxidant response maintains an appropriate redox state, thereby suppressing PRRSV-induced ROS damage. Nrf2 is a crucial factor in the cellular defense system, primarily regulating the expression of many genes involved in oxidative reactions, such as HMOX1, NQO1, GCLC, and GCLM [31]. The transcriptional activation of these enzymes is mainly achieved through the binding of Nrf2 to the antioxidant response element (ARE) on their 5′ promoters [70]. Studies have shown that the Nrf2 agonist 4-OI induces a cellular antiviral program against PRRSV, effectively inhibiting PRRSV replication through a type I interferon-independent mechanism [71]. Furthermore, it has been reported that natural compounds targeting the Nrf2 axis also show potential for anti-PRRSV therapy, such as the antimalarial drug artesunate, which inhibits PRRSV replication by activating the AMPK and Nrf2/HO-1 signaling pathways [72]. Liu et al. proved that xanthohumol significantly inhibits PRRSV proliferation and reduces virus-induced oxidative stress by activating the Nrf2-HO-1 pathway [62]. Biliverdin (BV), a downstream metabolite of HO-1, can inhibit PRRSV replication, and its secondary metabolite, bilirubin (BR), specifically mediates this anti-PRRSV activity through nitric oxide (NO)-dependent cGMP/PKG signaling pathway [73]. Taken together, our findings suggest that PAC-CDs are potent inducers of the Nrf2/ARE signaling pathway and may become a therapeutic agent for PRRSV infection.

The NLRP3 inflammasome is a key mediator of pyroptosis that is triggered by various viruses, including PRRSV, Zika virus, influenza A virus (IAV), and HCV [74–77]. When assembled, NLRP3 inflammasome facilitates the conversion of pro-caspase-1 into active caspase-1, which not only processes inactive cytokines IL-1β and IL-18 into their active forms but also cleaves GSDMD, resulting in the formation of a pore-forming N-terminal fragment. This fragment oligomerizes, causing a loss of cellular contents and leading to pyroptosis [78]. While this process mitigates pathogen replication, it can also trigger excessive inflammation in host tissues, leading to potential harm [63]. Moreover, ROS are important triggering factors for NLRP3 inflammatory vesicle activation, and Nrf2 activation can scavenge ROS and exert antioxidant effects, thus inhibiting the NLRP3 inflammatory vesicle-mediated pyroptosis pathway [79]. In recent years, some natural compounds and their derivatives have been shown to regulate the expression of NLRP3 inflammatory vesicles by activating Nrf2, and the inhibitory effect of Nrf2 on NLRP3 inflammatory vesicles has been verified in different diseases [32]. This study, in agreement with these findings, demonstrates that PAC-CDs significantly impaired the expression of NLRP3-mediated pyroptosis pathways such as NLRP3, ASC, Cleaved Caspase1, N-GSDMD, Cleaved IL-1β, and attenuated PRRSV infection. Furthermore, compared with the PRRSV-infected group, PAC-CDs also decreased the expression of pro-inflammatory cytokines IL-1β, IL-6, IL-8, and TNF-α. However, ML385 can upregulate the expression of both pyroptosis-related factors and inflammatory cytokines, reversing the protective effects of PAC-CDs. Additionally, PAC-CDs alleviated the level of oxidative stress in cells by activating Nrf2 and upregulating the expression of antioxidant genes, thereby inhibiting NLRP3 inflammasome-mediated pyroptosis and the release of inflammatory factors. Similarly, Xiang et al. reported that treatment with radix isatidis polysaccharide (RIP) can activate the antioxidant transcription factor Nrf2 to inhibit oxidative stress, thereby significantly reducing the expression of NLRP3 inflammasome and apoptosis-related proteins (including Bax, Caspase-3, and Caspase-9), ultimately mitigating Infectious bronchitis virus (IBV)-induced chicken interstitial nephritis [80]. Dimethyl fumarate improves erectile dysfunction in rats with bilateral cavernous nerve injury by activating the Nrf2/HO-1 signaling pathway to reduce ROS levels, promote SOD, and reduce 3-NT and MDA thereby inhibiting oxidative stress and NLRP3 inflammasome-mediated neural scotoma [41]. Collectively, this study shows that PAC-CDs can inhibit NLRP3 inflammasome-mediated pyroptosis in PRRSV-infected cells by activating the Nrf2/ARE pathway. These results suggest that PAC-CDs may effectively target the NLRP3 inflammasome and enhance the Nrf2 antioxidant response, offering a potential therapeutic strategy for reducing viral-induced pyroptosis and inflammation.

In this study, PAC-CDs show great potential to inhibit PRRSV infection. PAC-CDs exhibit lower toxicity and better water solubility, thus demonstrating superior biocompatibility and broader applications. We demonstrate that PAC-CDs can reduce the virus titer by approximately 4.59 × 10^3^-fold and effectively inhibit the internalization and replication stages of the PRRSV infection in both Mrac-145 and PAM cells. Subsequently, PAC-CDs triggered the activation of the Nrf2/ARE signaling pathway by enhancing antioxidant activity, reducing oxidative stress markers like MDA, and increasing the expression of antioxidant enzymes GSH and SOD. Furthermore, we found that PAC-CDs inhibited NLRP3 inflammasome-mediated pyroptosis and suppressed the expression of inflammation-associated factors to counteract virus-induced oxidative damage and inflammatory responses, and protected cells and tissues from damage caused by viral infection. This study provides strong support for the development of effective antiviral strategies and uncovers the potential application value of PAC-CDs against PRRSV infection.

## Supplementary Information

**Additional file 1. Synthesis and characterization of PAC-CDs.** (A) Photographs of PAC-CDs and water under sunlight and UV light (365nm) irradiation, respectively; (B) Photographs of PAC-CDs in different solutions on day 0 and day 7.

**Additional file 2.** **The primer sequences for the relative real-time PCR assay**. m means monkey, p means pig.

## Data Availability

Data sharing does not apply to this article as no datasets were generated or analyzed during the current study.
