# Supplementary figures and images for: Proanthocyanidins carbon dots inhibit PRRSV infection by activating Nrf2/ARE to regulate oxidative stress and NLRP3 inflammasome-mediated pyroptosis
Source: Vet Res. 2025 Oct 16;56:197. doi: 10.1186/s13567-025-01642-5 (PMC12533465; doi:10.1186/s13567-025-01642-5)

**
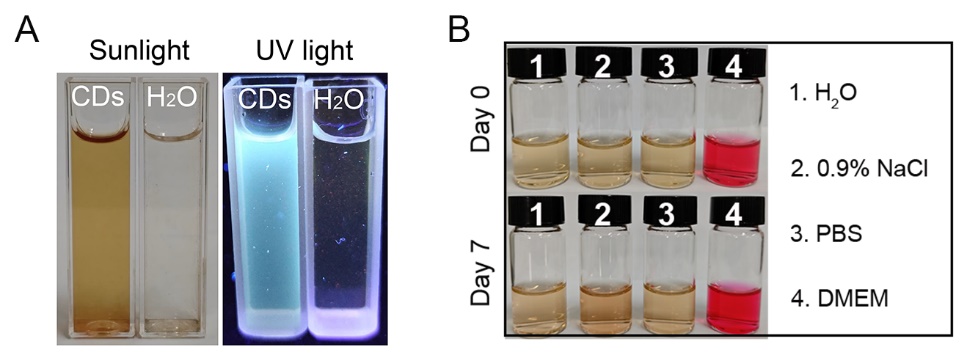
**

Supplement: Supplementary file 1 — Additional file 1. Synthesis and characterization of PAC-CDs. (A) Photographs of PAC-CDs and water under sunlight and UV light (365nm) irradiation, respectively; (B) Photographs of PAC-CDs in different solutions on day 0 and day 7. [file 13567_2025_1642_MOESM1_ESM.docx]
