# Supplementary material for: Proanthocyanidins carbon dots inhibit PRRSV infection by activating Nrf2/ARE to regulate oxidative stress and NLRP3 inflammasome-mediated pyroptosis
Source: Vet Res. 2025 Oct 16;56:197. doi: 10.1186/s13567-025-01642-5 (PMC12533465; doi:10.1186/s13567-025-01642-5)
Supplement: Supplementary file 2 — Additional file 2. The primer sequences for the relative real-time PCR assay. m means monkey, p means pig. [file 13567_2025_1642_MOESM2_ESM.docx]

**Additional file 2: The primer sequences for the relative real-time PCR assay.** m means monkey, p means pig.

| Primer | Nucleotide Sequence (5’-3’) |
| --- | --- |
| mGAPDH-F | CCTTCCGTGTCCCTACTGCCAA |
| mGAPDH-R | GACGCCTGCTTCACCACCTTCT |
| PRRSV-N-F | AAACCAGTCCAGAGGCAAG |
| PRRSV-N-R | TCAGTCGCAAGAGGGAAAT |
| mIL-1β-F | GGAAGACAAATTGCATGG |
| mIL-1β-R | CCCAACTGGTACATCAGCAC |
| mIL-6-F | AGAGGCACTGGCAGAAAAC |
| mIL-6-R | TGCAGGAACTGGATCAGGAC |
| mIL-8-F | CTGGCGGTGGCTCTCTTG |
| mIL-8-R | CCTTGGCAAAACTGCACCTT |
| mTNF-α-F | TCTGTCTGCTGCACTTTGGAGTGA |
| mTNF-α-R | TTGAGGGTTTGCTACAACATGGGC |
| pGAPDH-F | GATCCCGCCAACATCAAAT |
| pGAPDH-R | TTCACGCCCATCACAAACAT |
| pIL-1β-F | TCCACTTGGCGGTGATCA |
| pIL-1β-R | TCCACTTGGCGGTGATCA |
| pIL-6-F | CTGCTTCTGGTGATGGCTACTG |
| pIL-6-R | GGCATCACCTTTGGCATCTT |
| pIL-8-F | AGTTTTCCTGCTTTCTGCAGCT |
| pIL-8-R | TGGCATCGAAGTTCTGCACT |
| pTNF-α-F | CGTTGTAGCCAATGTCAAAGCC |
| pTNF-α-R | TGCCCAGATTCAGCAAAGTCCA |
| pNrf2-F | ATGCCCTCCTCTGCTACTT |
| pNrf2-R | CTTTCACGGTGGTCTTGGT |
| pHO-1-F | GGAGCACTCACAGCCCAACA |
| pHO-1-R | GTACAAGGACGCCATCACCAG |
| pNQO1-F | CCAAGTAGCCTCTTTGACCTA |
| pNQO1-R | ATGGATTTGCCCAAGTGAT |
| pGCLM-F | GGTTCATCTGTCCTTGGAGCAT |
| pGCLM-R | TTAAATCGGGCGGCATCAC |
| pGCLC-F | TCAGTAAGTCCCGATACGATT |
| pGCLC-R | TGATGAAGAGGTGAGCCAC |
| pTXNRD1-F | GGTGCTTGTGGTCTTTCCG |
| pTXNRD1-R | ACACGCTCATTGTCTTTGAT |
| pTNXIP-F | TCGGTCAGAGGCAATCACA |
| pTNXIP-R | CTTGGAGCCAGGGACACTA |
